# Chances and drawbacks of derepressed recombinant enzyme production in continuous cultivations with *Komagataella phaffii*

**DOI:** 10.3389/fbioe.2025.1523037

**Published:** 2025-03-10

**Authors:** Mihail Besleaga, Katharina Ebner, Anton Glieder, Oliver Spadiut, Julian Kopp

**Affiliations:** ^1^ Institute of Chemical, Environmental and Bioscience Engineering, Research Division Integrated Bioprocess Development, Vienna, Austria; ^2^ bisy GmbH, Hofstätten an der Raab, Austria

**Keywords:** derepressed feeding, chemostat, pseudohyphae, *Komagataella phaffii*, continuous cultivation

## Abstract

Utilizing *Komagataella phaffii* (*K. phaffii*) as a host, methanol-dependent fed-batch cultivations remain state-of-the-art for recombinant protein production. Recently, however, derepressible promoters have emerged as a valuable methanol-free alternative, especially for the expression of complex target proteins. In this study, we investigated the expression of a recombinant model enzyme (UPO) using a derepressible bi-directionalized promoter system in continuous cultivations. According to the literature, low growth rates required for derepression might result in pseudohyphae growth in chemostat cultivations with *K. phaffii*. This phenotype would be highly undesired as pseudohyphae growth is referred to decreasing productivity. Still, literature on derepressible promoter systems used in continuous cultivations is scarce. Hence, we aim to investigate pseudohyphae growth in a derepressible bi-directionalized promoter system. Several chemostats and a decelerostat screening were performed to identify the effect of the specific growth rate on pseudohyphae growth in continuous cultivations whilst monitoring the productivity of the recombinant target enzyme. Based on the experimental screening data, derepression was still achieved at a growth rate of 0.11 h^-1,^ whilst no pseudohyphae growth was observed. However, verifying these conditions for an extended timeframe of more than five residence times triggered pseudohyphae formation. Hence, the results of this study indicate that pseudohyphae growth in chemostats with derepressible promoter systems in *K. phaffii* is both growth-rate and time-dependent, thus limiting the potential of continuous cultivations for recombinant production of UPO. Despite the observed limitations, we still propose decelerostat cultivations as a proper screening tool to determine suitable production conditions in continuous systems for derepressed promotors.

## 1 Introduction

The recombinant protein market has grown steadily over the past years due to an increasing demand caused by the biotechnological industry (De Brabander et al., 2023; Rettenbacher et al., 2022; Bachhav et al., 2023). One of the key representatives of the recombinant protein market are enzymes (Barone et al., 2023). Recombinant enzymes initially became popular due to their widespread use in different domains of life and the significant role they play in multiple metabolic reactions (Spohner et al., 2015; Deveryshetty and Antony, 2021). An example thereof is the enzyme class of oxidoreductases (EC 1), which are present in all kingdoms of life (Kareem, 2020). Unspecific peroxygenases (UPO, EC 1.11.2.1) belong to the enzyme class of oxidoreductases that can catalyze non-activated C-H- and C=C- bonds as well as C-C bond cleavage while requiring only H_2_O_2_ for catalytic reactions (Ullrich et al., 2004; Kinner et al., 2021). However, due to the highly complex structures present in UPOs (disulfide bridges and heme moiety), soluble expression in prokaryotic hosts is challenging (Manta et al., 2019). As a result, eukaryotic hosts are commonly employed to produce UPOs recombinantly (Besleaga et al., 2024). The recombinant expression of UPOs was tested in various eukaryotic and prokaryotic hosts, whereas *Komagataella phaffii* (*K. phaffii*) was found to be the most favorable expression system (Hofrichter et al., 2022). Recently, for different short UPOs (*Abr*UPO, *Mwe*UPO-1), fed-batch bioreactor cultivations of recombinant *K. phaffii* strains were reported, which resulted in protein yields of around 0.7 g/L (Sánchez-Moreno et al., 2024; Schmitz et al., 2023).

Yeasts possess many advantages, such as fast growth, cheap media requirements, easy genetic manipulation tools, high cell density fermentations, the ability to perform post-translational modifications, and high secretory efficiency (Barone et al., 2023; Bernauer et al., 2021; Vijayakumar and Venkataraman, 2023; Karbalaei et al., 2020). *Komagataella phaffii* was implemented successfully for the recombinant production of various oxidoreductases, like peroxidases (EC 1.11.1.7 and 1.11.1.13) (Zhang et al., 2020; Pekarsky et al., 2018), peroxygenases (EC 1.11.2.1) (Gomez de Santos et al., 2023; Ebner et al., 2023; Tonin et al., 2021), catalases (EC 1.11.1.6) (Gómez et al., 2019), heme oxygenases (EC 1.14.99.3) (Mei et al., 2024) and cytochrome P450s (EC 1.14.14.1) (Hausjell et al., 2020; Garrigós-Martínez et al., 2021b).

Recombinant protein production in *K. phaffii* is often driven by the promoter of the major alcohol oxidase gene, P_*AOX1*_, which is inducible by methanol (Bernat-Camps et al., 2023; Bustos et al., 2022). The P_*AOX1*_ promoter shows tight regulation and strong induction by methanol as a sole carbon source (Bustos et al., 2022; Wurm and Spadiut, 2019), allowing a distinct separation between biomass and product formation. However, methanol imposes several disadvantages in industrial-scale production, like high flammability, cell toxicity, high oxygen consumption, and high heat of combustion (Dalvie et al., 2022; Pan et al., 2022; Wollborn et al., 2022). Due to the disadvantages of methanol, alternative induction systems are often required, especially for continuous cultivation (Nieto-Taype et al., 2020). For instance, for many carbon source-dependent promoters of methylotrophic yeasts, methanol-free promoter activation can be achieved by derepression (applying low feeding rates of repressing carbon source) (Vogl et al., 2020; Weinhandl et al., 2014). However, derepression of P_*AOX1*_ leads to low promoter activation, with recombinant protein expression levels of only 2%–4%, when compared to methanol-induced P_*AOX1*_ (Vogl et al., 2018; Yang and Zhang, 2018; Vogl et al., 2020). Alternative promotors, however, are well designed to be induced under carbon derepression, with one of them being the orthologous formate dehydrogenase promoter from *Hansenula polymorpha* (P_*DF*_, a commercial variant of the promoter) (Vogl et al., 2020). Induction of the P_*DF*_ by derepression (in *K. phaffii*) reached expression levels of approximately 75% compared to the methanol-induced P_*AOX1*_. Moreover, induction by derepression allows tight regulation of the promoter by a simple variation in feeding the repressing carbon source (Vogl et al., 2020).

Despite novel promotor systems available, many oxidoreductases suffer from low production yields due to their complexity (Ebner et al., 2023). A strategy to facilitate the expression levels is the co-expression of either chaperones or foldases (Irvine et al., 2014; Raschmanová et al., 2021). Here, for convenience reasons, a bi-directionalized promoter system can be implemented to express both proteins simultaneously from a single expression construct (Rajamanickam et al., 2017; Navone et al., 2021). Multiple chaperones are available for co-expression in *K. phaffii*, some of them, such as PDI (protein disulfide isomerase) and HAC1 (the unfolded protein response transcription factor), showed a significant improvement in protein expression and increase in product titers (Raschmanová et al., 2021). Moreover, a recent study also showed the feasibility of PDI co-expression for the production of the long archetype UPO (PaDa-I) in *K. phaffii*, as the product titer of recombinant UPO in the supernatant was doubled (Zhao et al., 2024).

When it comes to bioreactor-scale cultivation mode, the fed-batch is the current state-of-the-art for microbials to produce recombinant proteins (García-Ortega et al., 2019; Duman-Özdamar and Binay, 2021). However, continuous cultivation proved to be a powerful tool to achieve high space-time yields (Rahimi et al., 2019; de Macedo Robert et al., 2019; Nieto-Taype et al., 2020). Unfortunately, in *K. phaffii* chemostat cultivations (secreting recombinant human serum albumin) with specific growth rates (µ) set below 0.075 h^-1^, pseudohyphae growth was observed (Rebnegger et al., 2014). This was presumably caused by the upregulation of floculin (*FLO11*) gene (De et al., 2020). This is a major issue in recombinant protein production, as pseudohyphal growth is known to hinder protein secretion, leading to decreased productivity (Puxbaum et al., 2016). Interestingly, Garrigós-Martínez *et al.* recently employed a derepressible bi-directionalized promoter system to co-produce hCYP2C9 and the complementary reductase (hCPR) in chemostat cultivation with a D of 0.05 h^-1^ and did not report any pseudohyphae growth (Garrigós-Martínez et al., 2021b). This is surprising, considering the induction mechanism of derepressible promoters. Low dilution rates are required in chemostat cultivation, which is reported to trigger pseudohyphae growth. To our knowledge, no studies have further investigated pseudohyphal growth in chemostat cultivations using a derepressible promoter system for recombinant protein production with *K. phaffii*.

As literature is contradictory, this study aimed to shed more light on pseudohyphal growth in derepressed chemostat cultivations whilst expressing a recombinant target enzyme. For this purpose, we used a bi-directionalized promoter system for derepressible production of an unspecific peroxygenase (UPO, EC 1.11.2.1) and constitutive co-expression of a chaperon (PDI). This study aimed to mechanistically describe the impact of dilution rates on pseudohyphae growth, with the goal of determining stable production conditions in chemostat cultivations.

## 2 Materials and methods

### 2.1 Strain

The *K. phaffii* BSYBG11 strain was used to recombinantly produce an unspecific peroxygenase from the organism *Aspergillus novoparasiticus* (*Ano*UPO, EC 1.11.2.1). Different synthetic bi-directionalized promoter (BDP) systems were designed and constructed based on the bisy GmbH proprietary standard vector P_*BSY5Z*_, employing the strong derepressible/methanol inducible P_*DF*_ for the expression of the *Ano*UPO and different constitutive promoters (P_*UPP*_, P_*GAP*_, and P_*HHT*_) for expression of the protein disulfide isomerase (PDI). Protein disulfide isomerase (PDI) was selected as the chaperone for the co-expression as it was shown to have a beneficial impact on the recombinant production of various proteins (Raschmanová et al., 2021; Zhao et al., 2024) and help with disulfide bond formation (Zhao et al., 2024; Irvine et al., 2014; Inan et al., 2006). Moreover, a recent study demonstrated that PDI co-expression also improved the expression of an UPO (Zhao et al., 2024). Transformation of competent *K. phaffii* cells and UPO production strain selection via microscale screening and rescreening was done as described previously (Besleaga et al., 2024). The cells were kept at −80°C in 25% glycerol cryo stocks.

### 2.2 Bioreactor cultivations

All bioreactor cultivations started with the inoculation of the pre-culture medium with cryo stocks. The pre-culture medium was composed of 100 mL/L of sterilized 0.1 M potassium phosphate buffer pH 6.0, 13.4 g/L yeast nitrogen base without amino acids and with ammonium sulfate, 5 g/L (NH_4_)_2_SO_4_, 400 mg/L biotin, 20 g/L glycerol and Zeocin (end concentration 100 μg/mL). The biotin and yeast nitrogen base solution were prepared separately as a stock solution and filtered sterile through a 0.2 µm cutoff filter into a sterile flask. The pre-culture medium was inoculated with a fresh cryo stock, and it was incubated at 30°C, 230 rpm for 24 h in an Infors Multitron shaking incubator (Infors HT, Basel, Switzerland). After 24 h of incubation, the pre-culture was used to inoculate the batch medium (10% of the batch media volume). The batch medium composition was the following: 25 g/L sodium hexametaphosphate, 1.17 g/L CaSO_4_ · 2H_2_O, 18.2 g/L K_2_SO_4_, 14.9 g/L MgSO_4_ · 7H_2_O, 9.0 g/L (NH_4_)_2_SO_4_, 40.0 g/L glycerol and 4.5 mL/L of *Pichia* trace metal solution (PTM1, the composition of the trace metal solution was described by (Spadiut et al., 2014). The fed-batch cultivation was performed in a Minifors two bioreactor system (max. Working volume: 2 L; Infors HT, Basel, Switzerland). Process control and feeding were performed using EVE software (Infors HT, Bottmingen, Switzerland). The cultivation offgas was analyzed online using offgas sensors–IR for CO_2_ and ZrO_2_ based for Oxygen (Blue Sens Gas analytics, Herten, Germany). The pH was monitored using a pH-sensor EasyFerm Plus (Hamilton, Reno, NV, United States). During cultivations, pH was kept constant at 5.0 and was controlled with base addition only (12.5% NH_4_OH), while acid (10% H_3_PO_4_) was added manually, if necessary. The temperature was kept constant at 30°C. Aeration was carried out using a mixture of pressurized air and pure oxygen at two vvm to keep dissolved oxygen (dO_2_) above 30% at all times. The dissolved oxygen was monitored using a fluorescence dissolved oxygen electrode Visiferm DO (Hamilton, Reno, NV, United States). The fed-batch was started at the end of the batch phase, which was indicated by a drop in CO_2_ signal and a parallel increase in the dissolved oxygen. The feed medium for fed-batch cultivation consisted of 400 g/L glycerol supplemented with 12 mL/L PTM1.

For chemostat cultivations, similar equipment was used as described for fed-batch cultivation. The cultivation volume in the reactor was adjusted and maintained constant via an immersion tube connected to a bleed pump. During continuous cultivation, the stirrer speed and aeration rate were set to constant values. The feed medium composition for chemostat cultivation was the following: 37.5 g/L sodium hexametaphosphate, 1.17 g/L CaSO_4_ · 2H_2_O, 18.2 g/L K_2_SO_4_, 14.9 g/L MgSO_4_ · 7H_2_O, 4.5 g/L (NH_4_)_2_SO_4_, 40.0 g/L glycerol and 9.0 mL/L of PTM1. The dilution rates (D) were set based on the fed-batch results. For decelerostat cultivation, the starting D was set to 0.14 h^-1^ (85% µ_max_) to avoid cell washout. After five residence times, the D was decreased by 10% and maintained until a steady-state was achieved. The decrease in D was done until pseudohyphae cells were observed in the fermentation broth (analyzed with a microscope).

### 2.3 Process analytics

For analytics, fermentation broth samples were taken after inoculating the bioreactor, at the end of the batch phase, and then on a daily or bi-daily basis during the cultivations, depending on the chosen process conditions. Biomass concentration was determined optically, using optical density (OD_600_), and gravimetrically, via its dry cell weight (DCW). OD_600_ was measured using a Genesys 20 photometer (Thermo Scientific, Waltham, MA, United States). Due to the linear range of the used photometer, 0.1–0.8 absorption units, samples were diluted with deionized H_2_O to stay within that range. The DCW was determined by vortexing the sample of the fermentation broth, pipetting 1 mL of the sample solution in a pre-weighted 2 mL Eppendorf-Safe-Lock Tube (Eppendorf, Hamburg, Germany) and centrifugated at 14,000 rpm and 4°C for 10 min. After the centrifugation step, the supernatant was used immediately for at-line HPLC measurement, while the pellet was resuspended and washed with 1 mL of filtered 0.9% NaCl solution. After the resuspension of the biomass pellet with the 0.9% NaCl solution, the sample was centrifugated again, applying the same conditions as before. After the second centrifugation step, the supernatant was discarded, and the cell pellet was dried at 105°C for at least 48 h before weighing the pellet. Glycerol concentrations were determined via at-line HPLC (Thermo Scientific Waltham, MA, United States) using an Aminex column (HPX-87H Column, 300 · 7.8 mm, Bio-Rad, Hercules; CA, United States). The eluent was composed of 5 mM H_2_SO_4_, and the flow rate was set to 0.5 mL/min for 30 min. Glycerol standards were prepared in the range of 1–50 g/L. Chromatograms were analyzed using the Chromeleon Software (Dionex, Sunnyvale, CA, United States).

Microscopic analysis was used to detect pseudohyphae growth. Microscopy was performed with an Olympus CKX41 inverted microscope (Olympus Life Science, Tokyo, Japan) with an IX2-SLP phase contrast slider (Olympus Life Science, Tokyo, Japan) using a Canon EOS 250D (Canon, Tokyo, Japan) objective. Images were processed using ImageJ 1.52 days software (Schneider et al., 2012). Additionally, a reverse transcription-quantitative PCR (RT-qPCR) was performed to analyze the expression levels of the *FLO11*. The TAF10 gene was used as a reference gene for reliable RT-qPCR analysis (Besleaga et al., 2023).

### 2.4 Product analytics

To determine the total protein concentration in the supernatant, 1 mL of fermentation broth was pipetted in a 2 mL Eppendorf-Safe-Lock Tube (Eppendorf, Hamburg, Germany) and centrifuged for 10 min at 10,000 rpm at 4°C. After the centrifugation step, the supernatant was collected and analyzed according to the Bradford protocol (Bradford, 1976), while the pellet was discarded. The reaction mixture consisted of 200 µL of Bradford reagent solution mixed with 5 µL of the supernatant sample. The change in absorbance at 595 nm was measured after 10 min of incubation using a Tecan Infinite M200 PRO plate reader (Tecan, Männedorf, Switzerland).

Peroxidase activity in the supernatant was measured in a high-throughput 96-well format using ABTS as a substrate and a Tecan Infinite M200 PRO plate reader (Tecan, Männedorf, Switzerland). The reaction mixture, per well, consisted of 170 µL of ABTS solution (5 mM ABTS in 50 mM KH_2_PO_4_, pH 5), 10 µL of a sample (diluted with dH_2_O, if required), and 20 µL of H_2_O_2_ (final concentration 1 mM). After adding H_2_O_2_, the plate was immediately placed in the plate reader at 30°C, and the change of absorption at 420 nm was monitored for 2 min. The volumetric enzyme activity was calculated according to Humer et al. (Humer et al., 2020). Based on multiple measurements, the limit of detection for ABTS assay was determined as 0.05 U/L.

## 3 Results

### 3.1 Fed-batch cultivations

**FIGURE 1:**
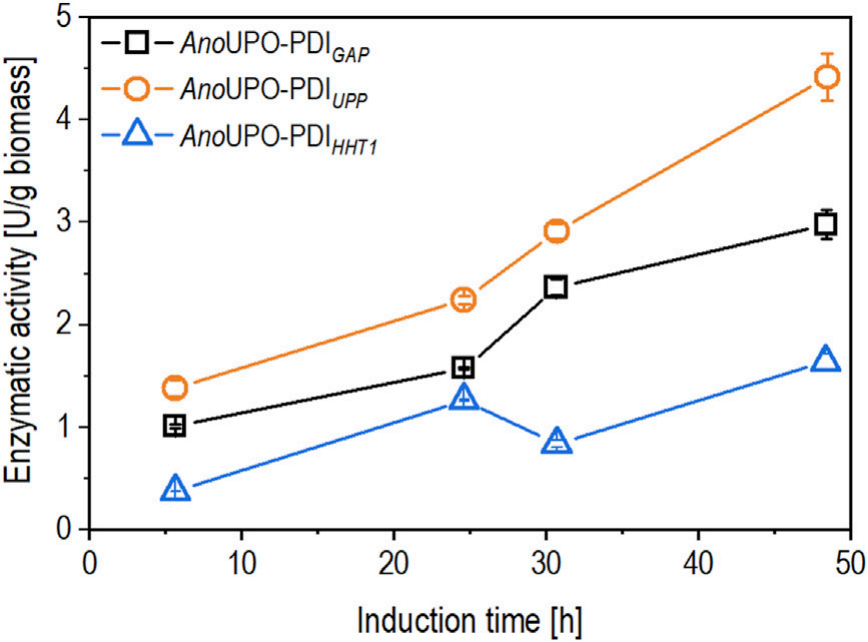
The time-dependent biomass-specific expression (in U/g biomass) was calculated in fed-batch cultivations. The induction was performed by derepression of the P_*DF*_ utilizing a growth rate of µ = 0.03 h^-1^. The same *Komagataella phaffii* strain was used in each cultivation, where the *Ano*UPO was always regulated by P_*DF*_ and the chaperon (protein disulfide isomerase, PDI) was regulated by P_*GAP*_ (open squares), P_*UPP*_ (open circles), and P_*HHT1*_ (open triangles). Data are represented as mean values of technical replicates ±standard deviation (n = 4).

Bi-directionalized promoters (BDP) allow concomitant expression of multiple genes of interest, thus improving the production of the recombinant target protein. In this study, co-expression of the chaperon protein disulfide-isomerase (PDI) was used to improve the secretory production of an unspecific peroxygenase (*Ano*UPO). After initial cloning efforts and screening in the microscale, fed-batch cultivations were performed to identify the best co-expression construct for the production of unspecific peroxygenase (*Ano*UPO) (Figure 1). In all constructs, P_*DF*_ was used for the expression of *Ano*UPO, while different constitutive promoters were used to co-express the PDI: glyceraldehyde-3-phosphate dehydrogenase (P_*GAP*_), a commercial variant of P_*GCW14*_ (P_*UPP*_) (Wang et al., 2019) and the *K. phaffii* histone promoter (P_*HHT1*_). For induction of UPO production via derepression of the P_*DF*_, the specific growth rate (µ) was set to 0.03 h^-1^, based on a previous study (Besleaga et al., 2024).

As shown in Figure 1, the biomass-specific enzyme activity revealed various production levels between the tested BDP after 6 hours of induction via derepression. The biomass-specific activity continuously increased throughout induction for the strains containing *Ano*UPO-PDI_*GAP*_ and *Ano*UPO-PDI_*UPP*_, while for *Ano*UPO-PDI_*HHT1*_ containing strain, the biomass-specific enzyme activity slightly decreased after 30 h of induction, followed by a later increase at the end of the cultivation. The strain *Ano*UPO-PDI_*UPP*_ showed the highest expression at the end of the cultivation and, therefore, was selected for further chemostat cultivations.

### 3.2 Initial chemostat experiments

**FIGURE 2:**
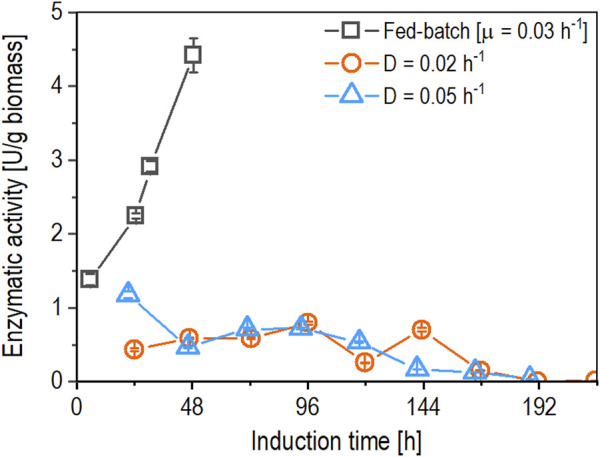
Comparison of time-dependent biomass-specific enzyme activity (U/g biomass) between fed-batch cultivation (open squares) operated at µ = 0.03 h^-1^ and chemostat cultivations operated at D of 0.02 h^-1^ (open circles) and 0.05 h^-1^ (open triangles) using a derepressible bi-directionalized promoter system. Data are represented as mean values of technical replicates ± standard deviation (n = 4).

Intriguingly, literature reports about the occurrence of pseudohyphal growth in chemostat cultivations with *K. phaffii* at high dilution rates are inconsistent. Although Rebnegger *et al.* observed pseudohyphal growth at growth rates below 0.075 h^-1^ (Rebnegger et al., 2014), Garrigós-Martínez et al. performed chemostat cultivations at growth rates of 0.05 h^-1^ for the derepressed production of recombinant enzymes and did not report any pseudohyphae formation (Garrigós-Martínez et al., 2021b; Garrigós-Martínez et al., 2021a). Hence, two initial chemostat cultivations with the *Ano*UPO-PDI_*UPP*_ were performed to test the behavior of the promotor system in continuous cultivation. Growth rates of 0.02 h^-1^ and 0.05 h^-1^ were selected since the previous study showed no significant difference in derepression from 0.02 h^-1^ to 0.05 h^-1^ (Besleaga et al., 2024). Furthermore, we wanted to test whether differences in pseudohyphae growth were present at given dilution rates and to determine the effect on biomass-specific enzyme activity (Figure 2).

When comparing the biomass-specific enzyme activity achieved in fed-batch and chemostat cultivations (Figure 2), both chemostats (operated at a dilution rate of 0.02 h^-1^ and 0.05 h^-1^) showed a nine-fold lower biomass-specific protein production. The biomass-specific enzyme activity in the chemostat operated at a dilution rate (D) of 0.02 h^-1^ started to fluctuate at 120 h, followed by a slight decrease after 144 h of induction, reaching values under the limit of detection (LOD) after 192 h of induction until the chemostat was stopped. In the case of the chemostat operated at D of 0.05 h^-1^, the biomass-specific expression decreased gradually after 96 h of induction, reaching values beneath the LOD after 192 h of induction until the chemostat was stopped.

**FIGURE 3:**
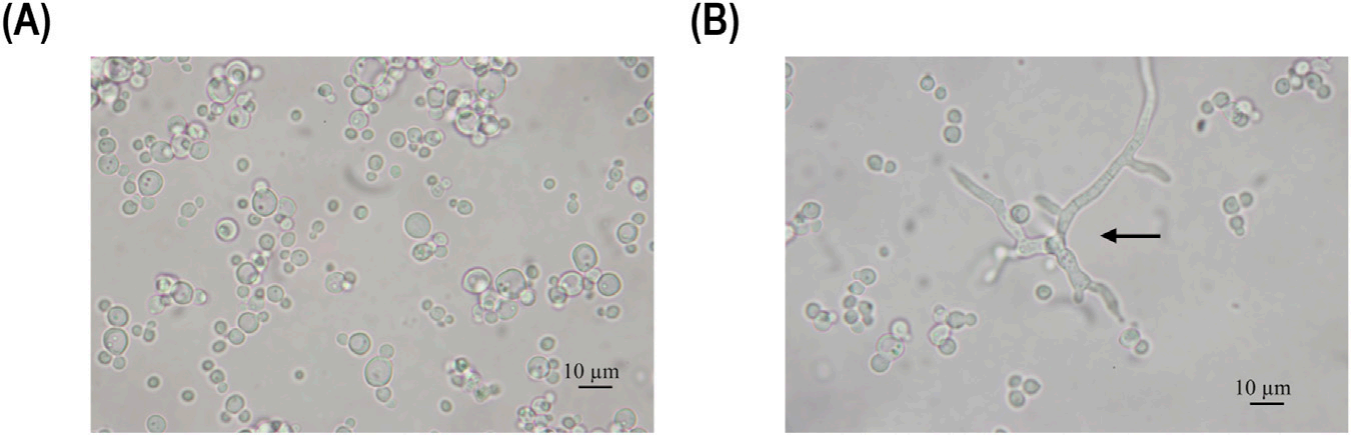
Microscopy pictures of the fermentation broth, sample taken at 68 h **(A)** and 115 h **(B)** of induction during the chemostat cultivation operated at a dilution rate D of 0.02 h^-1^. The arrow points to pseudohyphae cells.

The results of the chemostats indicate that the growth rates (µ) that resulted in the highest biomass-specific enzyme activity in fed-batch cultivation from the previous study (Besleaga et al., 2024) cannot be applied to chemostat cultivations. Using these growth rates in chemostat cultivations resulted in pseudohyphae growth, while no signs of pseudohyphae growth were observed in fed-batch cultivations. An example of pseudohyphae cells from the performed chemostats is shown in Figure 3.

**FIGURE 4:**
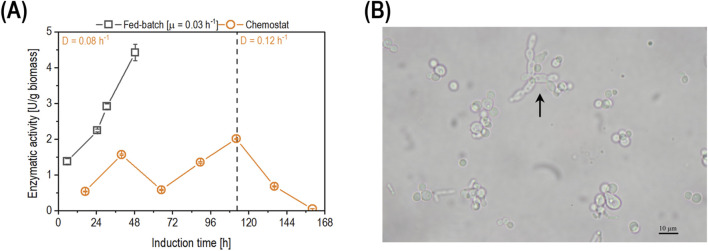
**(A)** Comparison of the time-dependent biomass-specific enzyme activity (U/g biomass) in fed-batch cultivation (open squares) and chemostat cultivation operated at D of 0.08 h^-1^ (open circles). The dashed line represents the time when the D was increased from 0.08 h^-1^ to 0.12 h^-1^. Data are shown as mean values of technical replicates ± standard deviation (n = 4). **(B)** Microscopy picture of the fermentation broth sample taken at 112 h of induction during the chemostat cultivation operated at D of 0.08 h^-1^. The arrow points to pseudohyphae cells.

As pseudohyphae growth and a decreased recombinant protein secretion were observed at dilution rates of 0.02 h^-1^ and 0.05 h^-1^, our results do not conform with previous literature reports for secretory enzyme production via derepressed promoter systems. Hence, a new dilution rate of 0.08 h^-1^ was investigated, targeting derepression while achieving no pseudohyphae growth (Figure 4).

As shown (Figure 4A), the biomass-specific enzyme activity in the chemostat fluctuated until the measured sample at 112 h of induction, where pseudohyphae growth was observed for the first time in this cultivation (Figure 4B). After 112 h of induction, the D was increased to 0.12 h^-1^ trying to stop pseudohyphae growth. However, the cells continued to display pseudohyphae despite the increase in µ. Productivity decreased simultaneously as promotor repression occurred. In order to identify a D at which no pseudohyphae growth would occur while maintaining the promoter at a derepressed state, a decelerostat cultivation was performed.

### 3.3 Decelerostat screening

**FIGURE 5:**
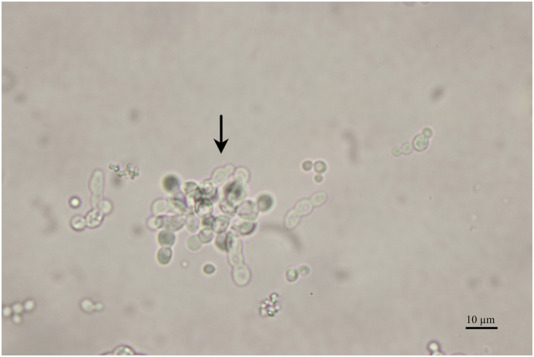
Microscopy image of the fermentation broth sample taken in steady-state from the chemostat cultivation operated at D of 0.09 h^-1^. The arrow points to pseudohyphae cells.

The starting D in the decelerostat cultivation was set to 85% of µ_max_ to avoid cellular washout. The D was decreased in 10% of µ_max_ intervals to monitor different dilution rates precisely. Each interval was kept steady for at least five residence times (RT, five-volume exchanges). The cultivation was gradually decreased from D = 0.14 h^-1^ until a D of 0.09 h^-1^ was achieved. No further decrease was performed since pseudohyphae growth was previously observed at D of 0.08 h^-1^ (Figure 5). The specific productivity of the decelerostat at different D is shown in Table 1.

**TABLE 1:** The table represents the biomass-specific enzyme activity (U/g biomass) at each steady-state for each interval during the decelerostat cultivation. The decrease in the D was performed stepwise (decrease of D by 10% of µ_max_), and each interval was maintained at least for five residence times). The starting D for the decelerostat was set to 0.14 h^-1^ (85% of µ_max_) to avoid cell washout. Data are represented as mean values ± standard deviation (n = 4).

D_set_ [h^-1^]	Enzymatic activity [U/g biomass]
0.14	beneath the LoD
0.12	beneath the LoD
0.11	0.43 ± 0.02
0.09	0.57 ± 0.01

As indicated in Table 1, no derepression was achieved at D of 0.14 h^-1^ and 0.12 h^-1^. However, when the chemostat was operated at a D of 0.11 h^-1^, the promoter was derepressed, and a biomass-specific enzyme activity during the steady-state of 0.43 ± 0.02 U/g biomass was reached. When the D was further decreased to 0.09 h^-1^, the biomass-specific activity (at steady-state) additionally increased to 0.57 ± 0.01 U/g biomass. However, pseudohyphae formation was already initiated at a D of 0.09 h^-1^ when the steady-state was achieved (Figure 5).

Based on the results of the performed decelerostat, the condition of D = 0.11 h^-1^ was selected for a verification experiment, as these conditions were the only determined ones, achieving promoter derepression whilst avoiding pseudohyphae formation.

### 3.4 Verification of determined chemostat conditions

**FIGURE 6:**
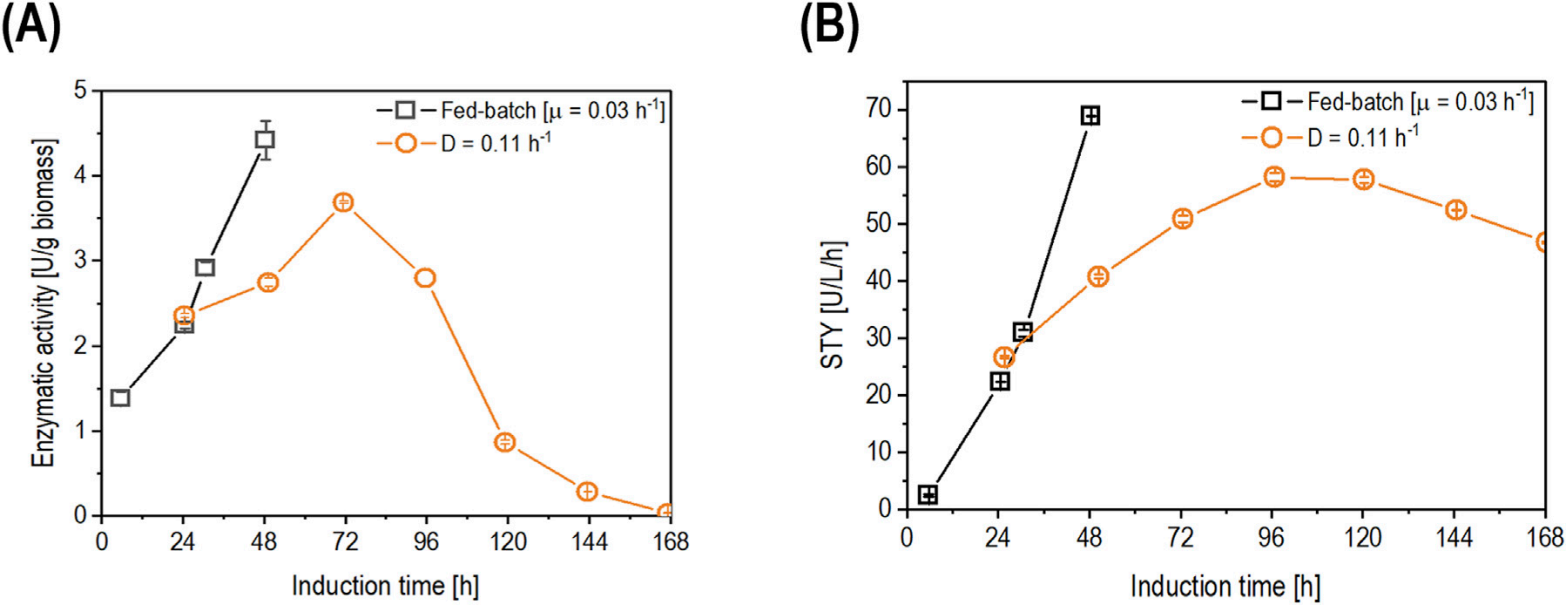
Comparison of fed-batch cultivation with µ of 0.03 h^-1^ (open squares) and chemostat cultivation (open circles) operated at D of 0.11 h^-1^ for **(A)** the time-dependent biomass-specific enzyme activity (U/g biomass) and **(B)** the space-time yield (U/L/h) Data are represented as mean values of technical replicates ± standard deviation (n = 4).

Due to the results of the decelerostat cultivation, production conditions for an elongated timeframe (= longer than five residence times) were performed in chemostat cultivation at a D of 0.11 h^-1^. Results were compared to the state-of-the-art cultivation mode, fed-batch with µ of 0.03 h^-1^ (Figure 6).

**FIGURE 7:**
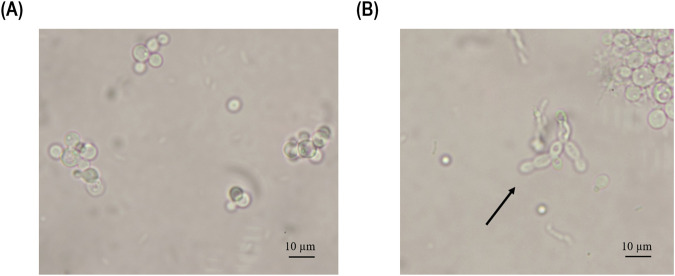
Microscopy pictures of the fermentation broth samples were taken at **(A)** 96 h and **(B)** 119 h of induction during the chemostat cultivation operated at D of 0.11 h^-1^. The arrow points to pseudohyphae cells.

After 24 h of induction, the biomass-specific enzyme activity in the chemostat showed a non-significant increase in productivity compared to the fed-batch cultivation (Figure 6A). Furthermore, the biomass-specific enzyme activity continued to increase in both cultivation modes. However, at 48 h of induction, the biomass-specific activity in fed-batch cultivation outperformed that in chemostat cultivation (Figure 6A). At 72 h of induction, the chemostat reached its highest value (3.69 U/g biomass) before starting to decrease over time, reaching values below the LoD (0.05 U/L) at 168 h of induction (Figure 6A). When comparing the STY (Figure 6B), the fed-batch cultivation reached 68.8 U/L/h after 48 h of induction, while the chemostat showed a comparable value of 58.1 U/L/h after 96 h of induction. Productivity started to decrease in the chemostat cultivation after 96 h of induction due to pseudohyphae growth, which was detected in the fermentation broth at 119 h of induction (Figure 7).

A reverse transcription-quantitative PCR (RT-qPCR) was performed to analyze the expression levels of *FLO11*, reported of initiating pseudohyphae (Table 2) (De et al., 2020).

**TABLE 2:** Reverse transcription-qPCR analysis of biomass samples taken from chemostat cultivation operated at D of 0.11 h^-1^ at the start of induction, 96 h of induction, and 119 h of induction. *TAF10* was used as a reference gene for analysis of the transcript levels of the *FLO11*.

Induction time [h]	Log2 relative transcript analysis of *FLO11* gene
0	0.20 ± 0.04
96	0.20 ± 0.06
119	0.96 ± 0.02

As shown in Table 2, samples were taken at the start of induction, 96 h and 119 h of induction. The first two samples showed results being within the boundaries of the RT-qPCR, while the samples taken at 119 h of induction showed approximately five-fold upregulation of the *FLO11* gene, verifying the trends regarding pseudohyphae formation observed in microscopy.

## 4 Discussion

In a pre-study, unspecific peroxygenase (*Ano*UPO) expression employing a derepressible bi-directionalized promoter combined with PDI co-expression was already described as a viable production strategy in fed-batch cultivations (Besleaga et al., 2024). In order to potentially improve space-time yields achieved within fed-batch production, continuous cultivations were evaluated in this study. However, considering the induction mechanism of the P_*DF*_, low growth rates are required for induction. Unfortunately, this triggers pseudohyphae growth in chemostat cultivations with *K. phaffii*, hindering extracellular recombinant protein production (Puxbaum et al., 2016; Aggarwal and Mishra, 2022). Pseudohyphae growth was well described in chemostats using a monodirectional promoter system for recombinant protein production when applying dilution rates below 0.075 h^-1^ (Rebnegger et al., 2016; Rebnegger et al., 2014). Contrary to these results, another study exercising recombinant enzyme production of a different target protein using a derepressible promoter system in chemostat cultivations with *K. phaffii* did not report any pseudohyphae growth at D = 0.05 h^-1^ (Garrigós-Martínez et al., 2021a). Additionally, for a derepressed bi-directionalized promoter system in chemostat cultivations, another study reported no pseudohyphae formation at a growth rate of 0.10 h^-1^ (Garrigós-Martínez et al., 2021b). Thus, limited knowledge with contradictory statements is available on pseudohyphae formation in *K. phaffii* chemostats with derepressed recombinant protein production. Therefore, this study aimed to investigate pseudohyphae growth in chemostat cultivations with a derepressible bi-directionalized promoter system and identify the potential and limitations.

Three different constructs were initially generated, expressing the chaperone protein disulfide isomerase (PDI) with different constitutive promoters (P_*GAP*_, P_*UPP*_, and P_*HHT1*_). The strain consisting of the *Ano*UPO-PDI_*UPP*_ construct showed the highest productivity compared to other cultivations (Figure 1) and was thus selected for further experiments. We hypothesize that the high production levels of the *Ano*UPO-PDI_*UPP*_ strains were achieved due to the strength of the P_*UPP*_, which provided more co-expressed chaperones to assist in *Ano*UPO folding.

For the initial chemostat cultivation, the dilution rate (D) was set to 0.05 h^-1^ to verify the findings reported in the literature (Garrigós-Martínez et al., 2021a). Still, the highest productivities in fed-batch cultivations for a similar construct were achieved at a µ set between 0.02 h^-1^ and 0.05 h^-1^ (Besleaga et al., 2024). Hence, additionally, we investigated a chemostat at a dilution rate of 0.02 h^-1^ to assess the transferability of fed-batch conditions to chemostat cultivation. As shown in Figure 2, the biomass-specific enzyme activity in the chemostats was nine-fold lower compared to the fed-batch cultivation. A decrease in productivity of 24% was observed in a different study using induction via derepression of P_*DF*_ for *Candida antarctica* lipase B (CalB) production when comparing chemostat to fed-batch cultivation (Garrigós-Martínez et al., 2021a). A reason for decreased productivity could be pseudohyphae growth, which was observed in the chemostats operated at D of 0.02 h^-1^ and 0.05 h^-1^ after approximately eight generations. The occurrence of pseudohyphae growth indicates a similar µ dependent behavior as previously published for untransformed *K. phaffii* strains and strains expressing a recombinant protein under the control of the constitutive P_*GAP*_ (Rebnegger et al., 2014; Rebnegger et al., 2016).

To avoid pseudohyphae growth and thus facilitate the recombinant UPO expression, the D was increased to 0.08 h^-1^, above the pseudohyphae threshold of 0.075 h^-1^ reported in literature (Rebnegger et al., 2014). Applying a D of 0.08 h^-1^, enzyme production increased approximately two-fold compared to initial chemostats (Figure 4A). Surprisingly, pseudohyphae growth still occurred (Figure 4B), despite the set D. Even though PDI-co expression was shown to improve the activity of the recombinant UPO (Besleaga et al., 2024), the authors hypothesize that constitutive PDI co-expression might increase the maintenance metabolism. This could lead to an adaptation mechanism of the pseudohyphae phenotype even at higher dilution rates than originally described in literature (Rebnegger et al., 2014). Once pseudohyphae growth was observed within the microscope, the D in the chemostat was increased to 0.12 h^-1^ for five residence times, trying to eliminate pseudohyphae growth. However, the pseudohyphae subpopulation only increased over process time, which is in accordance with the literature (Mösch, 2002). Mösch *et al.* attributed this to the advantages of pseudohyphal cells, such as a higher cell surface area, which allows for better carbon source assimilation, triggering pseudohyphae growth in budding yeast cells (Mösch, 2002). On top of pseudohyphae formation potentially hindering the secretion of *Ano*UPO, the derepressible promoter (P_DF_) was potentially repressed already at D = 0.12 h^-1^. Combining both effects, it is no surprise that *Ano*UPO productivity decreased at D = 0.12 h^-1^. Similar behavior was observed previously, indicating that pseudohyphae growth is a heritable phenotype (De et al., 2020), as upshifts in D did not eliminate pseudohyphae growth and it interfered with the secretion of the target protein (Puxbaum et al., 2016). Visual determination of pseudohyphae was taken as an indicator for process termination for ongoing experiments. Since pseudohyphae growth also occurred at D of 0.08 h^-1^, a decelerostat was performed to screen different dilution rates on the effect of pseudohyphae growth and enzyme expression. An accelerostat was not exercised as we obtained initial pseudohyphae formation to be irreversible.

In the decelerostat experiment, a gradual decrease of D was exercised (Table 1). No enzyme expression was observed at steady-states operated at D of 0.14 h^-1^ and 0.12 h^-1^, which can be attributed to promoter repression at screened growth rates. This would also be in accordance with the previously performed chemostat cultivation. When the D was decreased to 0.11 h^-1^, the promoter was derepressed, and the specific enzyme activity after a timeframe of five residence times was found at 0.43 ± 0.02 U/g biomass (Table 1). With a further decrease of D to 0.09 h^-1^, the specific enzyme activity increased by 32.5% (0.57 ± 0.01 U/g biomass, Table 1). However, the decelerostat was stopped after the steady state was achieved at D of 0.09 h^-1^, since pseudohyphae growth was detected, which was expected to decrease productivity over time (Puxbaum et al., 2016). Even though D of 0.09 h^-1^ showed the highest productivity in the decelerostat, the aim was to avoid pseudohyphae growth for follow-up experiments. Furthermore, initial results revealed that pseudohyphae formation triggers time-instable product formation. Therefore, a D of 0.11 h^-1^ was selected to establish a continuous process for derepressed *Ano*UPO production.

Performing the experiment at a D of 0.11 h^-1^, the *Ano*UPO levels in the chemostat reached similar values compared to the fed-batch cultivation during the first 24 h of the induction (Figure 6A). The productivity during the cultivation fluctuated, but samples taken in the first 100 h of induction showed no significant decrease in cell-specific *Ano*UPO production. After 119 h of induction, the specific enzymatic activity decreased promptly, and pseudohyphae cells could be observed via microscopy analysis (Figure 7B). To confirm pseudohyphae growth for this experiment, we evaluated the expression level of the gene reported to be responsible for pseudohyphae growth (*FLO11*) (De et al., 2020) using reverse transcribed qPCR (Table 2). The samples taken at the beginning of induction up until 96 h of induction showed results within the boundaries of the RT-qPCR, while the sample taken at 119 h of induction showed a five-fold upregulation of *FLO11*, indicating that pseudohyphal growth occurred within the given time frame (Table 2).

Evaluating pseudohyphae growth in the performed chemostat, we noticed that pseudohyphae appeared after 110 ± 10 h of induction in the cultivation with a D set to 0.11 h^-1^. For chemostats operated at D of 0.02 h^-1^ and 0.05 h^-1^, pseudohyphae growth was detected after 115 h of induction. The chemostat operated at D of 0.08 h^-1^ showed pseudohyphae growth after 112 h of induction. Still, the timeframe for pseudohyphae growth equaled for all of the cultivations at approximately 110 ± 10 h of induction. Hence, results indicate that pseudohyphae growth in chemostats might be time-dependent once µ is beyond a certain threshold. Further research with different strains, constructs, and target proteins is still required to confirm these findings. Still, the results of this study revealed pseudohyphae growth in derepressed chemostats to be dependent on µ and the induction time.

Despite the pseudohyphae formation at later time points, results showed that chemostat cultivations have a small operating window where they can be operated with derepression for at least 110 ± 10 h. The stable production phase, as well as the recombinant protein titer, could be potentially increased if a floculin-deficient strain is used to reduce pseudohyphae growth, as reported in a recent study (Rebnegger et al., 2024). Even though fed-batch cultivation outperformed chemostat cultivation for the production of the selected UPO, productivity in different cultivation modes might vary between different UPO production. The results of this study indicated a screening method to target stable operational levels for recombinant proteins. As shown for *Ano*UPO production in continuous cultivation, production could compete with fed-batch cultivations for 110 h of induction time despite pseudohyphae formation occurring in chemostats. Additionally, this study revealed that pseudohyphae phenotype formation is both µ- and time-dependent, aiding more knowledge on the topic of derepressed recombinant production in continuous cultivations with *K. phaffii*.

## Data Availability

The raw data supporting the conclusions of this article will be made available by the authors, without undue reservation.
